# Influenza-associated outpatient visits among children less than 5 years of age in eastern China, 2011–2014

**DOI:** 10.1186/s12879-016-1614-z

**Published:** 2016-06-10

**Authors:** Tao Zhang, Jun Zhang, Jun Hua, Dan Wang, Liling Chen, Yunfang Ding, Shanshan Zeng, Jing Wu, Yanwei Jiang, Qian Geng, Suizan Zhou, Ying Song, A. Danielle Iuliano, Carolyn M. Greene, Jeffrey McFarland, Genming Zhao

**Affiliations:** Department of Epidemiology, School of Public Health, Fudan University, P.O. Box 289, No.138 Yi Xue Yuan Road, Shanghai, 200032 China; Key Laboratory of Public Health Safety, Ministry of Education, Shanghai, China; Collaborative Innovation Center of Social Risks Governance in Health, Shanghai, China; Suzhou Center for Disease Prevention and Control, Suzhou, China; Suzhou University Affiliated Children’s Hospital, Suzhou, China; Centers for Disease Control and Prevention, Atlanta, GA USA

**Keywords:** Influenza, Outpatient visit, Influenza-like illness, Children, China

## Abstract

**Background:**

The disease burden of influenza in China has not been well described, especially among young children. The aim of this study was to estimate the incidence of outpatient visits associated with influenza in young children in Suzhou, a city of more than 11 million residents in Jiangsu Province in eastern China.

**Methods:**

Influenza-like illness (ILI) was defined as the presence of fever (axillary temperature ≥38 °C) and cough or sore throat. We collected throat swabs for children less than 5 years of age with ILI who visited Suzhou University Affiliated Children’s Hospital (SCH) outpatient clinic or emergency room between April 2011 and March 2014. Suzhou CDC, a national influenza surveillance network laboratory, tested for influenza viruses by real-time reverse transcription-polymerase chain reaction assay (rRT-PCR). Influenza-associated ILI was defined as ILI with laboratory-confirmed influenza by rRT-PCR. To calculate the incidence of influenza-associated outpatient visits, we conducted community-based healthcare utilization surveys to determine the proportion of hospital catchment area residents who sought care at SCH.

**Results:**

The estimated incidence of influenza-associated ILI outpatient visits among children aged <5 years in the catchment area of Suzhou was, per 100 population, 17.4 (95 % CI 11.0–25.3) during April 2011-March 2012, 14.6 (95 % CI 5.2–26.2) during April 2012-March 2013 and 21.4 (95 % CI: 10.9–33.5) during April 2013-March 2014. The age-specific outpatient visit rates of influenza-associated ILI were 4.9, 21.1 and 21.2 per 100 children aged 0- <6 months, 6- <24 months and 24- <60 months, respectively.

**Conclusion:**

Influenza virus infection causes a substantial burden of outpatient visits among young children in Suzhou, China. Targeted influenza prevention and control strategies for young children in Suzhou are needed to reduce influenza-associated outpatient visits in this age group.

## Background

Influenza virus infection remains an important cause of medically attended respiratory illness in young children. In the United States and Europe, many studies have documented substantial morbidity and mortality in children and economic loss to their families associated with influenza illness in this age group [1–5]. Recent prospective surveillance studies in the United States showed that the average number of children 0–59 months of age hospitalized due to influenza per year is 0.9 per 1,000 children [6]. Further, outpatient visits attributable to influenza among children less than five years of age are even more common, with a range of 10 to 250 times more common than hospitalizations [6–8]. Data from Asian middle and high income countries (Thailand, Hong Kong, Singapore, and Japan) have shown that the estimated burden of human influenza illness is greater than previously appreciated and similar to that of the United States and European countries [9].

In developing countries, sentinel hospital surveillance can provide data to describe influenza epidemiology and seasonality, characterizing the circulating strains of influenza virus to guide vaccine development, and monitoring influenza pandemics. However, these surveillance systems can seldom be used to define the burden of influenza, because they are conducted in referral hospitals, where the catchment population is difficult to define and the health-seeking patterns may not be representative of the overall population [10]. Some attempts have been made to adjust medical facility-based disease burden estimates by conducting health utilization surveys to assess the proportion of disease not captured because of healthcare seeking behaviors [10–12]. Accurate estimates of disease burden are needed to establish public health priorities for resource allocation and to inform prevention, control and treatment policies.

China is the world’s most populous country with 64 million children less than 5 years old in 2012 [13]. However, the disease burden of influenza virus infection in Chinese children has not been well described. Although that China CDC’s technical guidelines recommend vaccination for young children, current influenza vaccine coverage is low, with estimates of coverage among those aged less than 5 years ranging from 1.2 to 37.7 % [14–16]. Well-designed population-based surveillance and research studies that yield representative data on the burden of influenza virus infection are needed to guide decision making on vaccination programs. Although Yu et al. reported that influenza caused substantial hospitalization burden in central China, especially among young children [17], there is no publication describing the burden of influenza-associated outpatient visits among young children in China. In April 2011, we initiated a hospital-based influenza surveillance system for children <5 years of age in the city of Suzhou in Jiangsu Province. We also conducted multiple phone-based healthcare utilization surveys in the same area from January 2012 to January 2014. For this study, we used healthcare utilization patterns in the catchment area of our surveillance hospital to estimate the incidence of outpatient visits associated with influenza in children less than 5 years of age during three influenza seasons.

## Methods

### Study site

**Fig. 1:**
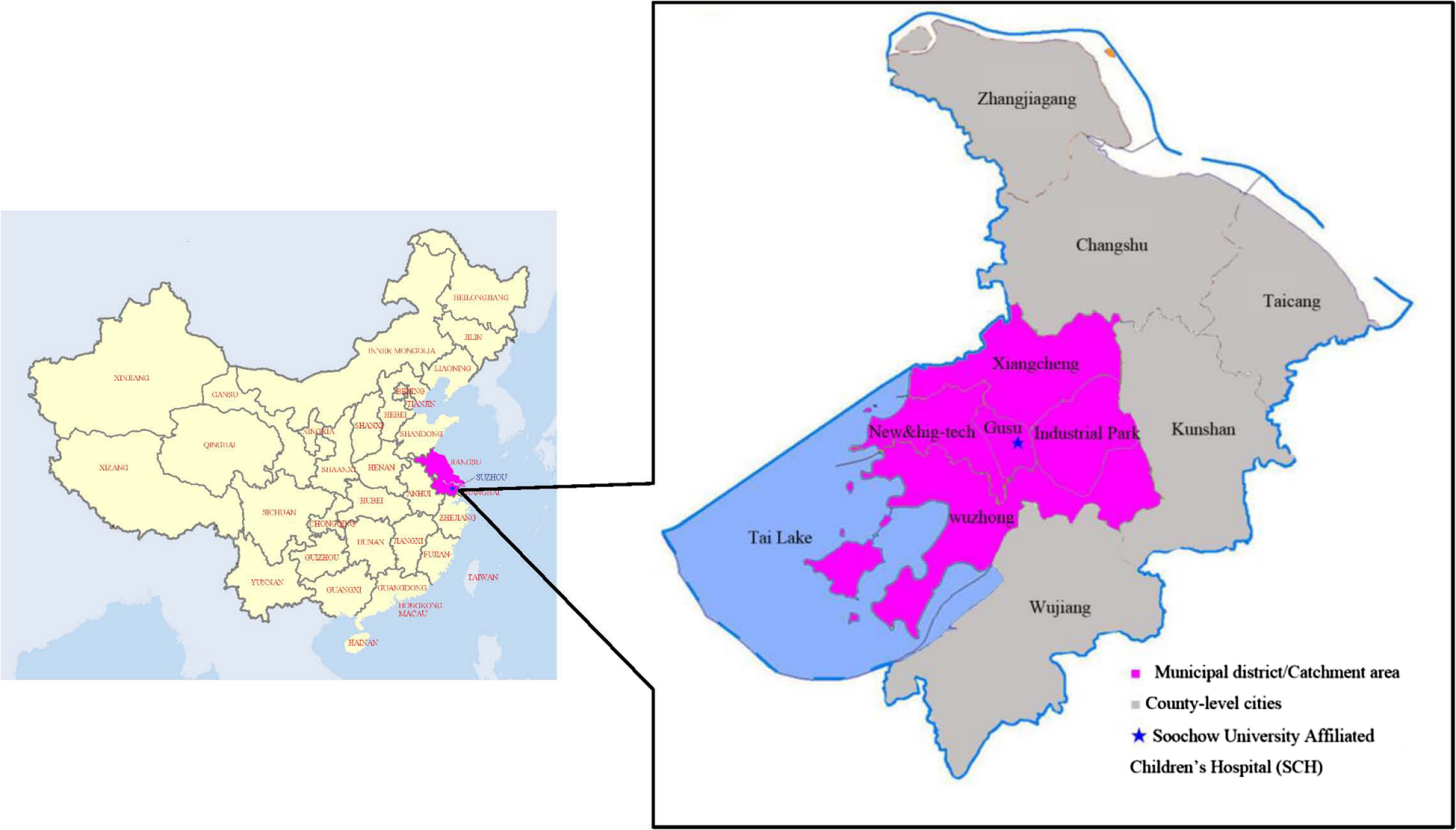
Map of the jurisdiction of Suzhou and the catchment area of Soochow University Affiliated Children’s Hospital (SCH)

This study was conducted in Suzhou, a major city located in Jiangsu Province, eastern China. Suzhou is one of the largest cities of the Yangtze Delta; it has an area of 8,488 square-kilometers and a population of approximately 12 million people, half of whom are migrants. The gross domestic product (GDP) per capita, calculated for the resident population only, was 18,000 US dollars in 2012 [18, 19]. Based on 2011 estimates in Suzhou, the population of children <5 years old was 239,528 Suzhou residents and 288,670 migrants (data from the immunization program database). Suzhou consists of 5 municipal districts and 5 county-level cities (Fig. 1).

Suzhou University Affiliated Children’s Hospital (SCH), located in the central area of Gusu District, is the only tertiary children’s hospital in Suzhou and it serves almost all young children in Suzhou. Hospital records from the influenza surveillance system at SCH from 2011 to 2014 demonstrated that 84.9 % of influenza-like illness (ILI) cases who sought care at SCH resided in the municipal districts (Table 1). Thus we defined the catchment area of SCH as the 5 municipal districts in Suzhou (Fig. 1).

**Table 1:** Regional source of enrolled influenza-like illness (ILI) cases <5 years of age from surveillance at Suzhou University Affiliated Children’s Hospital, 2011–2014

Area	Resident population^b^ N (%)	Enrolled ILI cases^c^ N (%)
Municipal district^a^	90,756 (43.4)	3,173 (84.9)
Counties	118,520 (56.6)	458 (12.2)
Wujiang	25,082 (12.0)	232 (6.2)
Kunshan	23,503 (11.2)	165 (4.4)
Changshu	29,222 (14.0)	31 (0.8)
Zhangjiagang	28,178 (13.5)	23 (0.6)
Taicang	12,535 (6.0)	7 (0.2)
Outside Suzhou	-	108 (2.9)

^a^Municipal district comprises Gusu district, New and High-tech district, Wuzhong district, Xiangcheng district and Industrial Park (Fig. 1)

^b^Resident population (refers to people living in Suzhou for more than 6 months) was obtained from the Suzhou immunization platform in 2010, which covered at least 95 % of the population of children in Suzhou

^c^Data from influenza surveillance for ILI in children <5 years old at Suzhou Children’s Hospital during 2011–2014

### Hospital-based surveillance for ILI

From April 2011 to March 2014, we conducted surveillance for influenza-like illness (ILI) among children less than 5 years old. We defined ILI as the presence of fever (axillary temperature ≥38 °C) and cough or sore throat. Inflamed or red pharynx on examination by the physician was used to identify sore throat in young children. Eligible children were those who resided in a district within the defined SCH catchment area for longer than 6 months and presented to SCH’s outpatient clinic or emergency department within 7 days of symptom onset. Among these, children eligible for sample collection were those who presented within 3 days of symptom onset. We provided training on our ILI surveillance system including the enrollment process to a team of 20 attending physicians among the approximately 50 physicians who rotate through the outpatient and emergency departments for a period of 1–3 months each year. Every month, we randomly selected 1–3 physicians who were serving in the outpatient and emergency departments to enroll patients from Monday to Friday. The selected physicians obtained a throat swab from every eligible patient whose parent or guardian provided informed consent. The full-time investigators or physicians also administered standard questionnaires to collect information related to demographics, clinical features and healthcare seeking behaviors associated with the illness. Each day, the full-time investigators recorded the total number of patients < 5 years old who visited our selected attending physicians from all districts and counties, the number eligible for ILI surveillance from all districts and counties, and the number enrolled and sampled for influenza virus testing.

### Laboratory tests for influenza virus

Throat swabs were placed in tubes with viral transport media (Youkang Technology Co., Beijing, China), stored at −80 °C and transported by cold chain to the National Influenza Surveillance Network of Suzhou Center for Disease Control and Prevention (CDC) every two days. Specimens were stored at −80 °C until tested for influenza virus. Viral RNA was extracted using High Pure Viral RNA Kit (Roche, Shanghai, China) according to the manufacturer’s instructions. For influenza virus testing, we performed real-time reverse transcription polymerase chain reaction (rRT-PCR) using influenza virus A/B dual fluorescent quantitative RT-PCR kit (BioPerfectus Technology Co., Jiangsu, China). The influenza virus A subtype identification was performed using influenza virus A historical H1N1/A H3N2/A 2009pdmH1N1) real time RT-PCR kit (ZJ Bio-Tech Co., Shanghai, China).

### Community-based healthcare utilization survey

We conducted seven phone-based community healthcare utilization surveys (HUS) in January 2012, October 2012, January 2013, April 2013, August 2013, November 2013 and January 2014. Stratifying the area by municipal district and county, we randomly sampled children who were <5 years of age and registered in the Suzhou child immunization platform. This registry includes all resident children and those migrant children who have been immunized two or more times. More than 95 % of resident children in Jiangsu Province receive all vaccinations within the Expanded Program on Immunization (EPI), while more than 85 % of migrant children in the province receive all EPI vaccinations (http://www.jswst.gov.cn/wsyw/snxw/2014/04/24152245125.html). In each HUS, we used a probability proportionate to size sampling strategy to sample approximately 1 % of the population from 5 age groups (<1 year, 1- <2years, 2- < 3-years, 3- < 4 years, and 4- <5 years). After a child under 5 years of age was identified, a trained interviewer attempted to reach the parent or guardian by phone to obtain oral informed consent and, if consent was given, administer a standardized healthcare utilization survey by telephone. Within the Suzhou immunization database, 97 % of all children have a guardian who has at least one telephone number. Information collected included whether the child had developed symptoms of ILI in the month prior to the interview; if they had sought care for the illness, where they sought care and if they had sought care at SCH specifically.

### Estimating burden of disease

Population data were obtained from the immunization program database for the years 2011–2014. We assumed that the outpatient visits to the attending physicians selected to enroll cases each day were representative of all outpatient visits at SCH. Thus the total number of ILI visits to SCH was extrapolated as follows: ILI proportion among outpatient visits to selected physicians over a period of time multiplied by the total number of outpatient visits in SCH over the same time period. The total number of influenza positive ILI visits in SCH (i) was calculated as:

$$ \mathrm{i}=\mathrm{influenza}\kern0.5em \mathrm{positive}\kern0.5em \mathrm{proportion}\kern0.5em \mathrm{of}\kern0.5em \mathrm{enrolled}\kern0.5em \mathrm{I}\mathrm{L}\mathrm{I}\kern0.5em \mathrm{cases}\kern0.5em \times \kern0.5em \mathrm{total}\kern0.5em \mathrm{I}\mathrm{L}\mathrm{I}\kern0.5em \mathrm{cases}\kern0.5em \mathrm{in}\kern0.5em \mathrm{S}\mathrm{C}\mathrm{H} $$

And the total number of influenza-associated ILI outpatient visits in the catchment area (t) was calculated as:

$$ \mathrm{t}=\mathrm{i}\times \frac{total\kern0.5em  number\kern0.5em  of\kern0.5em  medically\kern0.5em  attended\kern0.5em ILI\kern0.5em  cases\kern0.5em  in\kern0.5em  the\kern0.5em HUS\kern0.5em  surveys}{number\kern0.5em  of\kern0.5em ILI\kern0.5em  cases\kern0.5em  seeking\kern0.5em  care\kern0.5em  in\kern0.5em SCH\kern0.5em  in\kern0.5em  the\kern0.5em HUS\kern0.5em  surveys} $$

Thus, we estimated the annual incidence of influenza-associated outpatient visits in the catchment area as:

$$ \frac{``t"\kern0.5em  in\kern0.5em a\kern0.5em  year}{The\kern0.5em  population\kern0.5em  of\kern0.5em  the\kern0.5em  catchment\kern0.5em  area}\times 100\% $$

The time periods were defined as April 2011 to March 2012, April 2012 to March 2013, and April 2013 to March 2014. We used data from the first HUS (January 2012) in the estimation period of April 2011-March 2012, data from the second HUS (October 2012) and third HUS (January 2013) in the estimation period of April 2012-March 2013, and data from the fourth HUS (April 2013), fifth HUS (August 2013) sixth (November 2013) and seventh HUS (January 2014) in the estimation period of April 2013-March 2014.

We used the Wilson confidence intervals for binominal distribution to estimate the 95 % confidence intervals (CIs) of the incidence rate for influenza-associated ILI [20].

## Results

### ILI surveillance in the outpatient clinic

**Fig. 2:**
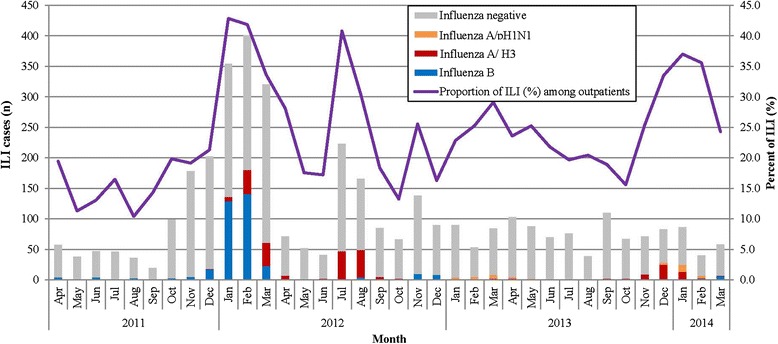
Seasonal distribution of enrolled influenza-like illness (ILI) cases positive for influenza among children <5 years of age and percent of ILI among all outpatient visits for children <5 years of age in Suzhou University Affiliated Children’s Hospital, Suzhou, April 2011-March 2014

From April 2011 to March 2014, we identified 11,817 (24.1 %) ILI cases within 49,058 outpatient visits among children less than 5 years old living in the SCH catchment area. The percentage of ILI among all outpatient visits peaked in January-March 2012, July- August 2012, February- March 2013 and December 2013 to February 2014 (Fig. 2). Among the 11,817 identified ILI cases, the male to female ratio was 1.27:1, and the median age was 19.5 months (IQR: 10.5–36.0).

### Seasonality of influenza

In this study, we enrolled 3,968 ILI cases for the 3 years of surveillance, and among these, we collected throat swabs from 3,571 (90.0 %) ILI case-patients, of which 603 (16.9 %) tested positive for an influenza virus. Among the 603, 266 (44.1 %) tested positive for influenza A (233 for subtype A/H3 and 33 for subtype A/pH1N1) and 337 (55.9 %) tested positive for influenza virus B. During April 2011 to March 2012, 383 (23.3 %) of 1,646 ILI cases tested positive for influenza virus, and 82 (21.4 %) were positive for influenza virus A (all for subtype A/- H3) and 301 (78.6 %) were positive for influenza virus B. During April 2012 to March 2013, 140 (12.9 %) of 1,088 ILI cases tested positive for influenza virus and among these, 114 (81.4 %) tested positive for influenza virus A (102 for subtype A/- H3 and 12 for type A/- pH1N1) and 26 (18.6 %) tested positive for influenza virus B. From April 2013 to March 2014, 80 (9.6 %) of 836 ILI cases tested positive for influenza virus, and 70 (87.5 %) were positive for influenza virus A (49 for subtype A/- H3, 21 (30.0 %) for subtype A/- pH1N1) and 10 (12.5 %) were positive for influenza virus B. There were two peaks of influenza activity, the first was from December 2011 to March 2012 with seasonal influenza B dominating, and the second was July-August 2012 with seasonal influenza A H3 predominating. From January 2013 to March 2014, seasonal influenza A H3 and pandemic influenza A H1N1 co-circulated (Fig. 2).

### Healthcare utilization surveys

In the 7 HUS surveys, we sampled a total of 25,505 children less than 5 years of age in Suzhou. We reached the parents or guardians of 14,366 children within our sample, for a connection rate of 56.3 %. Among parents and guardians reached, 12,722 agreed to be interviewed, for a response rate of 88.6 %. Finally, we excluded 220 questionnaires with missing information, leaving a total of 12,502 valid questionnaires. Of the 12,502 children, 6431 (51.4 %) were from the municipal districts and 6071 (48.6 %) were from the surrounding counties. Among those surveyed, the male to female ratio was 1.1:1 and the median age was 24.0 months (IQR: 13.0–42.0). There was no significant difference in age and gender between municipal districts and counties.

Of the 6431 children in municipal districts, 475 (7.4 %) parents self-reported that their child had symptoms of ILI in the one month prior to interview. Among these, 401 (84.4 %) had sought care for the ILI episode with a healthcare provider. Of these, 175 (43.6 %) had sought care at SCH. Of the 6071 children from the surrounding counties, 350 (5.8 %) parents reported that their children had an ILI episode in the one month prior to survey. Among those who sought healthcare from the surrounding counties, only 17(6.1 %) had sought care at SCH. This finding was statistically significantly lower than that of municipal districts (*χ*^2^ = 68.5, *p* < 0.001). The occurrence of ILI and the healthcare seeking behaviors were similar among survey periods, gender and age group (Table 2).

**Table 2:** Healthcare seeking behaviors among children with influenza-like illness (ILI) from healthcare utilization surveys in Suzhou, China, 2012–2014

	Municipal districts^a^							Counties^a^						
	No. total enrolled	ILI in preceding one month^b^		Sought care for ILI^c^		Sought care at SCH^d^		No. total enrolled	ILI in preceding one month^b^		Sought care for ILI^c^		Sought care at SCH^d^	
		n	%	n	%	n	%^c^		n	%	n	%	n	%
Gender														
Male	3364	264	7.8	223	84.5	103	46.2	3137	183	5.8	145	79.2	7	4.8
Female	3067	211	6.9	178	84.4	72	40.4	2934	167	5.7	132	79.0	10	7.6
Age														
0- < 6m	587	19	3.2	14	73.7	9	64.3	573	13	2.3	8	61.5	0	0.0
6- < 12m	976	77	7.9	69	89.6	43	62.3	860	44	5.1	33	75.0	1	3.0
12- < 24m	1555	129	8.3	112	86.8	49	43.8	1375	65	4.7	55	84.6	3	5.5
24- < 36m	1226	91	7.4	73	80.2	31	42.5	1133	70	6.2	55	78.6	4	7.3
36- < 48m	1095	98	8.9	84	85.7	30	35.7	1092	89	8.2	65	73.0	5	7.7
48- < 60m	992	61	6.1	49	80.3	13	26.5	1038	69	6.6	61	88.4	4	6.6
Total	6431	475	7.4	401	84.4	175	43.6	6071	350	5.8	277	79.1	17	6.1

^a^Municipal district comprises Gusu district, New and High-tech district, Wuzhong district, Xiangcheng district and Industrial Park. Counties include Changshu, Taicang, Zhangjiagang, Wujiang and Kunshan. (Fig. 1)

^b^Number of children identified as having symptoms of ILI in the one month prior to the HUS interview with their parent or guardian

^c^Among those with ILI in the past one month

^d^Among those with ILI who sought care in the past one month. (SCH = Suzhou University Affiliated Children’s Hospital)

### Estimating the incidence of influenza-associated ILI outpatient visits

We estimated that the incidence of influenza-associated medically attended ILI among children aged <5 years in the catchment area of SCH (defined as the 5 municipal districts of Suzhou) was 17.4 (95 % CI 11.0–25.3) per 100 population during April 2011-March 2012, 14.6 (95 % CI 5.2–26.2) per 100 population during April 2012-March 2013 and 21.4 (95 % CI: 10.9–33.5) per 100 population during April 2013-March 2014 (Table 3).

**Table 3:** Incidence of influenza-associated influenza-like illness (ILI) in the municipal district of Suzhou, April 2011-March 2014 (95 % CI)

Parameters	Apr 2011-Mar 2012	Apr 2012-Mar 2013	Apr 2013-Mar 2014
Proportion of ILI in outpatient visits (%)	26.1	24.8	23.2
Proportion of influenza in ILI (%)	23.3	13.3	9.5
Total No. of outpatient visits at SCH^a^	301,278	319,086	343,927
Estimated total no. of outpatient visits for ILI at SCH^b^	72,244 (61,292–83,196)	81,782 (71,199–92,365)	85,676 (79,117–92,235)
Estimated total no. of influenza-associated ILI at SCH^c^	8,956 (5,657–13,018)	7,172 (2,557–12,872)	10,965 (5,579–17,132)
Resident population of children <5 years in the catchment area of SCH^d^	99,622	111,663	123,207
Proportion of ILI who sought care at SCH (%)^e^	51.6	44.0	41.5
Incidence of influenza-associated ILI per 100 person-year	17.4 (11.0–25.3)	14.6 (5.2–26.2)	21.4 (10.9–33.5)

^a^Total number of outpatient visits at SCH from catchment area was estimated by applying the proportion of ILI cases from catchment area to the total number of outpatient visits at SCH; ^b^Estimated by multiplying the month-specific number of outpatient visits at SCH by the month-specific proportion of ILI in outpatient visits; month specific data used as influenza has a seasonal epidemic pattern; ^c^Estimated by multiplying the month-specific estimated number of outpatient visits for ILI at SCH by the month-specific proportion testing positive for influenza virus; month specific data used as influenza has a seasonal epidemic pattern; ^d^Resident population in the catchment area (refers to people living in Suzhou for more than 6 months) was obtained from Suzhou immunization program database;^e^ We used data from 1^st^ HUS (Jan 2012) in the estimation period of Apr 2011-Mar 2012, data from 2^nd^ HUS (Oct 2012) and 3^rd^ HUS (Jan 2013) in the estimation period of Apr 2012-Mar 2013, and data from 4^th^ HUS (Apr 2013), 5^th^ HUS (Aug 2013) 6^th^ (Nov 2013) and HUS 7^th^ (Jan 2014) in the estimation period of Apr 2013-Mar 2014

The age-specific outpatient visit rates of influenza-associated ILI were 4.9, 21.1 and 21.2 per 100 person-years in children aged 0- <6months, 6- <24months and 24- <60months, respectively (Table 4).

**Table 4:** Age-specific incidence of influenza-associated influenza-like illness (ILI) in the municipal district of Suzhou, Apr 2011-Mar 2014

Age group (month)	Resident population of children <5 years in the catchment area of SCH	Observed influenza positive cases at SCH	Estimated total no. of influenza -associated ILI at SCH (95 % CI)	Proportion of ILI who sought care at SCH (%)	influenza-associated ILI outpatient visits per 100 population (95 % CI)
Apr 2011-Mar 2012					
0- <6	11,057	19	420 (265–611)	66.7	5.7 (3.6–8.3)
6- < 24	31,729	146	3,224 (2,036–4,686)	56.5	18.0 (11.4–26.1)
24- < 60	56,836	241	5,319 (3,360–7,732)	47.4	19.7 (12.5–28.7)
Total	99,622	406	8,956 (5,657–13,018)	51.6	17.4 (11.0–25.3)
Apr 2012-Mar 2013					
0- <6	12,393	10	487 (173–875)	50.0	7.9(2.8–14.1)
6- < 24	35,564	72	3,514 (1,253–6,307)	43.2	22.9 (8.2–41.1)
24- < 60	63,706	65	3,170 (1,130–5,689)	44.1	11.3 (4.0–20.2)
Total	111,663	147	7,172 (2,557–12,872)	44.0	14.6 (5.2–26.2)
Apr 2013-Mar 2014					
0- <6	13,674	2	252 (128–394)	80.0	2.3 (1.2–3.6)
6- < 24	39,241	37	4,660 (2,371–7,281)	52.6	22.6 (11.4–35.3)
24- < 60	70,292	48	6,052 (3,079–9,457)	30.6	28.1 (14.3–44.0)
Total	123,207	87	10,965 (5,579–17,132)	41.5	21.4 (10.9–33.5)
Apr 2011-Mar 2014					
0- <6	37,124	31	1,160 (568–1,881)	64.3	4.9 (2.4–7.9)
6- < 24	106,533	255	11,398 (5,660–18,275)	50.8	21.1 (10.5–33.8)
24- < 60	190,834	354	14,542 (7,570–22,879)	35.9	21.2 (11.0–33.4)
Total	334,491	640	27,102 (13,799–43,035)	43.6	18.6 (9.5–29.5)

## Discussion

This is the first study in China to estimate the incidence of laboratory-confirmed influenza-associated ILI outpatient visits in children younger than 5 years old using surveillance data for ILI in the outpatient setting combined with data from healthcare utilization surveys. The estimated influenza-associated ILI outpatient visits among children aged <5 years in Suzhou ranged from 14.6 to 21.4 per 100 population over the three seasons of the study period. The outpatient visit rate of influenza-associated ILI was lowest among infants <6 months of age.

The rate of outpatient visits for influenza among young children in our study was similar to that observed in other regions of southern Asia [9]. In a study from Bangladesh which used similar estimating methods, the outpatient visits for influenza-associated ILI varied from 10 to 35 per 100 person-years among children during the 2008–2010 seasons [12]. The incidence of influenza-associated ILI outpatient visits in our study in 2012–2103 (14.6 per 100 population) was also similar to that observed in rural India in 2001–2004 in children aged less than 3 years with acute respiratory infection and influenza (14 per 100 person years) [21]. However, the incidence in our study was higher than that in Thailand (14 per 1000 population in 2003–2004 for all age groups) [22]. In Thailand, the effective catchment population of the surveillance sites was calculated using health care utilization data for cases with pneumonia, which may have led to an underestimation of the percentage of people seeking care for ILI in facilities other than the surveillance sites. Our results were also higher than those in a study from Singapore which used the indirect model estimating method [23]. Estimates of influenza-associated ILI outpatient visit rates may vary by country or region due to both methodological differences and the actual variation in influenza activity by season and population [24].

The incidence of influenza-associated ILI outpatient visits in 2011–2012 was higher than that in 2012–2013, consistent with the trend of influenza-associated SARI during these two years in south China [25]. Although several studies have shown that disease due to influenza A virus is significantly more severe than that due to influenza B virus [5, 26], other studies have shown that influenza B virus can lead to a high burden of serious influenza disease in children [17]. In our study, although we didn’t evaluate the severity of influenza disease by type, the influenza-associated outpatient visit rate was greater in the season when influenza virus type A predominated or type A and B were co-circulating. The variation in influenza-associated outpatient visit rates by season and region demonstrates the importance of collecting surveillance data for several years to accurately estimate the burden of influenza in Suzhou.

The average annual rates of outpatient visits attributable to influenza among children less than 5 years of age were approximately 20 to 30 times higher than the hospitalization rates in this age group [17], which is consistent with studies from the US and Finland [3, 6]. As expected, hospitalized children represent only a small fraction of all children infected with influenza. The bulk of influenza-related disease burden is seen in the outpatient setting, where influenza can lead to very high consultation rates and frequent complications [27]. Each year, outpatient visits due to influenza in children, similar to hospitalizations, incur substantial medical costs and indirect costs in terms of parental absence from work [28–30].

The outpatient visit rate of influenza-associated ILI was lowest among infants <6 months of age. This finding is consistent with prior studies from the US [6]. Maternal antibodies may confer some protection against influenza illness among infants <6 months of age. Further, young infants, who are less mobile and may spend less time away from home, may have less opportunity for infection. In addition, influenza infections in young infants may not cause respiratory symptoms as frequently as in older children and adults [31]. Fortunately, vaccination is available for children ≥6 months, who have substantially higher rates of outpatient visits due to influenza-associated ILI [7]. Prevention methods among infants ≤6 months of age include immunizing pregnant women and caregivers of young infants.

Our study has several limitations. First, we estimated influenza-associated ILI outpatient visit incidence from the catchment area of one tertiary hospital, which may not be representative of the entire Suzhou District. Second, we used the same case definition for ILI to screen and collect specimens for all children <5 years of age. Infants <6 months of age with influenza may not present with the same respiratory symptoms as older children and adults, which would lead to an under-estimate of the burden of influenza in this age group. Third, findings from patients enrolled from the two to three selected outpatient and emergency department physicians each month may not be generalizable to all of SCH’s patients with ILI. To assess this potential bias, we compared the proportion of ILI visits among all outpatient visits seen by our selected physicians with the proportion seen by all other SCH outpatient and emergency department physicians. The proportions were similar (33.5 % vs. 31.6 %, *p* = 0.20). Fourth, although we know that the migrant population comprises about 50 % of the total population in Suzhou, we could not clearly differentiate the migrant children from the resident children in this study. Thus, we do not know if the incidence of influenza-associated ILI outpatient visits was higher among migrant or resident children. However, the HUS surveys suggested that there were no significant differences among resident and migrant populations in Suzhou with respect to ILI incidence (6.5 % vs. 6.7 %) and proportions seeking healthcare for ILI (81.7 % vs. 85.5 %). Fifth, given the challenge of collecting nasopharyngeal swabs in young children, this study only collected throat swabs. Nasopharyngeal swabs have been shown to have a greater sensitivity for influenza B virus, and thus it is possible that the use of throat swabs alone led to an underestimation of the burden of outpatient visits due to influenza B [32, 33].

Despite these limitations, this study is the first to provide estimates of the incidence of influenza-associated outpatient visits among young children in China. Although there is an extensive influenza surveillance system in China, previously, we were unable to estimate the burden of influenza-associated outpatient visits. In our study, we used data from hospital-based influenza surveillance, laboratory tests and healthcare utilization surveys to estimate influenza-associated ILI outpatient visits in the SCH catchment area. Our study provides the foundation for other regions in China to conduct similar estimates.

## Conclusions

There is a substantial burden of influenza-associated outpatient visits among young children in Suzhou, China. Influenza is a vaccine-preventable disease. Targeted influenza prevention and control strategies for young children are needed to reduce influenza-associated outpatient visits in this age group.

## Abbreviations

CDC, centers for disease control and prevention; CIs, confidence intervals; EPI, expanded program on immunization; GDP, the gross domestic product; HUS, healthcare utilization surveys; ILI, Influenza-like illness; rRT-PCR, real-time reverse transcription-polymerase chain reaction assay; SCH, Suzhou University affiliated children’s hospital
